# Functional jejunal interposition, a reconstruction procedure, promotes functional outcomes after total gastrectomy

**DOI:** 10.1186/s12893-015-0032-2

**Published:** 2015-04-15

**Authors:** Xuewei Ding, Fang Yan, Han Liang, Qiang Xue, Kuo Zhang, Hui Li, Xiubao Ren, Xishan Hao

**Affiliations:** Department of Gastrointestinal Oncology, National Clinical Research Center for Cancer, Tianjin Cancer Institute and Hospital, Tianjin Medical University, Huanhuxi Road, Ti-Yuan-Bei, Hexi District, Tianjin, 300060 P. R. China; Department of Pediatrics, Division of Gastroenterology, Hepatology and Nutrition, Vanderbilt University Medical Center, Nashville, TN USA; Department of Immunology, Key Laboratory of Cancer Prevention and Therapy, National Clinical Research Center for Cancer, Tianjin Cancer Institute and Hospital, Tianjin Medical University, Tianjin, P. R. China; Department of Laboratory Animal Science, Peking University Health Science Center, Beijing, P. R. China

**Keywords:** Gastric cancer, Gastrectomy, Inflammation, Motility, Reconstruction procedure

## Abstract

**Background:**

Functional jejunal interposition (FJI) has been applied as a reconstruction procedure to maintain the jejunal continuity and duodenal food passage after total gastrectomy in patients with gastric cancer. The purpose of this study was to evaluate clinical efficacy of the FJI procedure by comparing the functional outcomes of FJI to Roux-en-Y after total gastrectomy in gastric cancer patients, and investigate physiologic mechanisms by which FJI exerts beneficial outcomes in beagles.

**Methods:**

Patients with stage I-IV gastric cancer without metastasis and recurrence one year after surgery were enrolled in this retrospective study. Seventy one patients received FJI and seventy nine patients received Roux-en-Y after total gastrectomy. We evaluated the nutritional status at three and twelve months and incidence of complications up to twelve months after surgery. Beagles receiving sham operation, FJI, or Roux-en-Y after total gastrectomy were sacrificed forty eight hours postoperatively. Beagles were gavaged with active carbon for evaluating the intestinal transit rate. Intestinal tissues from the duodenojejunal anastomosis were collected for examining interstitial cells of Cajal (ICC), inflammation, and apoptosis.

**Results:**

Compared to the bodyweight before surgery, the bodyweight loss at three and twelve months after surgery in patients receiving FJI was significant less than that in patients with Roux-en-Y. Patients with the FJI procedure showed significant increase of blood hemoglobin and total protein, compared to those at one month after surgery, and the prognostic nutrition index scores at three and twelve months after surgery. The incidence rates of post-operative complications, including reflux esophagitis, dumping syndrome, and Roux-en-Y syndrome were decreased in patients with FJI. Compared to beagles receiving Roux-en-Y, more ICC in the intestinal submuocsa, less intestinal epithelial cell apoptosis, and decreased inflammation in serosal side of the intestine were found in the FJI group. The intestinal transit rate in FJI group was lower than that in Roux-en Y group, indicating that FJI benefits food storage.

**Conclusion:**

The FJI procedure promotes nutritional recovery and decreases post-operative complications in gastric cancer patients after total gastrectomy, which may be through ameliorating intestinal inflammation and damage and reducing ICC loss to preserve food reservoir function and intestinal motility.

## Background

Total gastrectomy, a common operation for patients with gastric cancer, is associated with a variety of early and late postoperative complications, leading to mal-absorption, reflux oesophagitis, dumping and weight loss. To prevent these post-gastrectomy complications, more than fifty gastric reconstruction procedures have been applied after total gastrectomy in patients with gastric cancer. Currently, the conventional Roux-en-Y procedure is the most widely used procedure [1]. Most of these procedures have the beneficial effects on improving the necessity and efficacy of gastric substitution and duodenal food-passage. However, these reconstruction procedures have limitations for preserving the reservoir function and digestion after total gastrectomy, which result in some common problems such as dietary restriction, early satiety, postprandial fullness, vomiting, heartburn, and diarrhea [2-4]. Studies from randomized control clinical trials have suggested that establishing a jejunal reservoir to create a gastric reservoir function could maintain a low speed of the food passage in the upper intestinal tract and retain the food storage volume, which might improve the post-operation food capacity and nutritional status [5-8]. In our previous clinical practice, we developed a reconstruction procedure, functional jejunal interposition (FJI), which is characterized by maintaining jejunal continuity and duodenal food passage [9]. Our previous clinical studies evaluated effects of six reconstruction procedures on post-operative outcomes and nutritional status after total gastrectomy in patients with gastric cancer, including jejunal continuity (FJI, Braun, modified Braun I and II) and jejunum transection (Roux-en-Y and jejunal interposition) [10]. Among these six procedures, FJI was the only procedure to combine the benefits of jejunal continuity and maintaining the duodenal food passage, which plays a key role in improving nutritional status after total gastrectomy. This clinical observation indicates that FJI may serve as a potential application to improve the quality of patient’s life after total gastrectomy.

In this report, we first evaluated the functional outcomes of FJI to the Roux-en-Y procedure with jejunum transection in after total gastrectomy in patients with gastric cancer. Furthermore, we performed a preliminary animal study to investigate the physiologic mechanisms by which FJI exerts beneficial effects after total gastrectomy in beagles. Our results suggest that FJI promotes nutritional recovery and decreases post-operative complications in patients after total gastrectomy. The FJI’s beneficial effects are associated with preserving the reservoir function and maintaining intestinal motility. Thus, FJI may serve as a recommended surgical method for total gastrectomy.

## Methods

### Patients

This clinical research project was approved by the Ethics Committee of Tianjin Cancer Institute and Hospital, Tianjin Medical University, P.R. China, and registered in Tianjin Medical University Cancer Institute and Hospital clinical trial center designated by the Chinese State’s Food and Drug Administration. We used AJCC cancer staging manual, sixth edition (2002) to define the cancer stage for patients in this study. Patients with histological diagnosis of gastric adenocarcinoma stage I- IV were enrolled in this retrospective study. These patients did not have metastasis and recurrent tumor after one year of surgery. Patients received total gastrectomy at Tianjin Medical University Cancer Institute and Hospital, Tianjin, China. Patients were randomly selected for FJI or Roux-en-Y after total gastrectomy. Seventy-one patients received FJI and seventy-nine patients received Roux-en-Y in this study. Detailed patient information was shown in Table 1. There is no significant difference about age, sex, and disease stage between FJI and Roux-en-Y groups.

**Table 1:** **Patient information**

	**Roux-en-Y**	**FJI**	***P value***
Male	59	40	p > 0.05
Female	20	31	p > 0.05
Age (mean, years)	58	59	p > 0.05
Stage I-II	14	13	p > 0.05
Stage III-IV	65	58	p > 0.05
**Pathology**	**Roux-en-Y**	**FJI**	***P value***
Tubular adenocarcinoma	27	7	P < 0.05
Papillary carcinoma	7	5	P > 0.05
Mucinous adenocarcinoma	12	6	P > 0.05
Carcinoma mucocellulare	8	20	P < 0.05
Poorly	22	28	p > 0.05
differentiated adenocarcinoma			
Diffused carcinoma	3	5	p > 0.05

*P values* are computed using Wilcoxon rank sum test for continuous data (age) and Fisher’s exact test for categorical data (sex, stage, pathology).

The exclusion criteria for this study were: 1) patients older than 75-year old; 2) patients with liver cirrhosis, cardiovascular diseases, or diabetes; 3) tumors occupied the whole stomach or linitis plastica; 4) advanced cases with obvious invasion to perigastric organ or tissue; 5) cases with positive resected margins confirmed pathologically during operation.

### Operation procedures for patients

**Figure 1:**
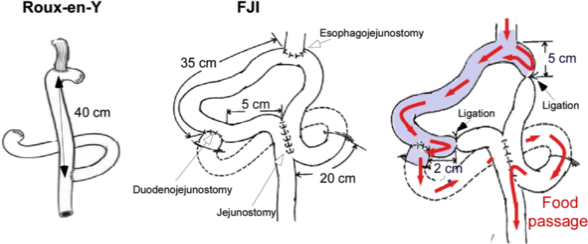
Schematic models of the reconstruction procedures after total gastrectomy performed for patients and for beagles.

Specialized gastrointestinal surgeons with more than 10-year experience performed all surgeries in this study. After the standard total gastrectomy and lymph node dissection, FJI or Roux-en-Y procedure was performed for patients (Figure 1).

To perform the FJI procedure, an end-to-side esophagojejunostomy was performed at 40 cm anal to Treitz’s ligament. Then, an end-to-side duodenojejunostomy was created at the efferent limb at 35 cm distal to the esophagojejunostomy. After these steps, a side-to-side jejunostomy at 5 cm distal to duodenojejunostomy and 20 cm distal to Treitz’s ligament was created. Finally, two jejunal ligations were made at 5 cm oral to esophagojejunostomy and 2 cm distal to duodenojejunostomy. The tension applied for ligation was to stop the food transit, but not to induce regional jejunal tissue necrosis.

For the Roux-en-Y procedure, after the standard total gastrectomy, Roux-en-Y procedure was performed by constructing the end-to-side jejunojejunostomy at 40 cm from esophagojejunal anastomosis.

### Assessment of post-operative nutritional status and complications

Patients were followed-up every 3 months after surgery and up to 12 months post-operatively. Patients were examined regularly by endoscopy, CT scan and ultrasound. Body weight, blood hemoglobin and total protein levels, and prognostic nutritional index (PNI, the score of PNI = 10 × serum albumin (g/100 ml) + 0.005 × total lymphocytes count/mm^3^ in peripheral blood) were used to evaluate postoperative nutritional status. All therapeutic factors other than alimentary reconstruction, such as chemoradiation and other medications, were excluded at the time of investigation.

The incidence of postoperative complications, including reflux esophagitis, dumping syndrome, and Rou-en-Y syndrome, were evaluated. The diagnosis of reflux esophagitis was made by a combination of clinical symptoms, including heartburn, acid dyspepsia, regurgitation, and chest pain, and objective testing with upper endoscopy. Endoscopic examination was assessed using the Los Angeles Classification; one (or more) mucosal break within the tops of two mucosal folds with the size < 5 mm (grade A) and > 5 mm (grade B), and continuous mucosal break between the tops of two or more mucosal folds which involve < 75% (grade C) and > 75% (grade D) of the circumference. Patients who had the above symptoms and the endoscopic assessment at the level of ≥ grade C were diagnosed as reflux esophagitis.

Dumping syndrome and Roux-en-Y syndrome were evaluated based on patients’ medical history using questionnaire and laboratory tests. The questionnaire was asked by an experienced nurse being blinded by the type of the procedure. Dumping syndrome was diagnosed based on symptoms, including abdominal cramps, nausea, diarrhea, tachycardia, sweating, flushing, and dizziness after eating, and oral glucose tolerance test. Roux stasis syndrome was diagnosed based on symptoms, including nausea, vomiting of food but not bile, epigastric fullness, and postprandial pain.

### Operation procedures for beagles

All animal experiments were performed according to protocols approved by the Institutional Animal Care and Use Committee at Peking University Health Science Center, Beijing, P. R. China. Beagles (Vital River Laboratories, Beijing, P. R. China) were maintained in a controlled environment at 21° under a 12-h dark/light cycle. All beagles used in this study were at six month old and body weights were between 7 kg and 10 kg.

Beagles were randomly selected for Sham operation (5 beagles), FJI (10 beagles) or Roux-en-Y (7 beagles) after total gastrectomy, performed by the same specialized gastrointestinal surgeon (Figure 1). Beagles were fasting for 12 hours before anesthetized using 2% Pentobarbital Sodium Salt. After the standard total gastrectomy, The Roux-en-Y procedure was performed by constructing the end-to-side jejunojejunostomy at 40 cm from esophagojejunal anastomosis. The ends of the Roux and Y limb were closed (Figure 1). To perform FJI procedure, an end-to-side esophagojejunostomy was performed at 40 cm anal to Treitz’s ligament. Then, an end-to-side duodenojejunostomy was created at the efferent limb at 35 cm distal to the esophagojejunostomy, followed by a side-to-side jejunostomy at 5 cm distal to duodenojejunostomy and 20 cm distal to Treitz’s ligament. Finally, 2 jejunal proper ligations were made at 5 cm oral to esophagojejunostomy and 2 cm distal to duodenojejunostomy (Figure 1). After surgery, beagles were given 5% glucose and 0.9% saline (1000 ml/day) with restriction of oral food intake.

### Measurement of intestinal transit rate

At 47 hours postoperatively, beagles were anesthetized using 2% Pentobarbital Sodium Salt and administered 50 ml of 10% active carbon in water to the esophagojejunal junction through a gavage tube. Beagles were sacrificed 1 hour after gavage and the active carbon migration distance in the small intestine was measured. The intestinal transit rate was calculated as: the active carbon migration distance/the total small intestinal length.

### Tissue section preparation

The intestine tissues were collected 48 hours after operation, including 5 cm oral and 5 cm anal to the site of the duodenojejunal anastomosis in beagles receiving FJI, 5 cm oral and 5 cm anal to the site of the jejunojejunostomy in beagles receiving Roux-en-Y, and jejunal tissues from beagles receiving sham operation. Tissue samples were fixed in 10% formalin for preparing and paraffin-embedded intestinal tissue sections.

### Analysis of intestinal inflammation

Paraffin-embedded tissue sections were stained with hematoxylin and eosin for light microscopic examination to assess intestinal inflammation. Samples were examined by a pathologist blinded to the treatment condition.

### Immunohistochemistry

Antigen retrieval of formalin-fixed sections was performed using the Antigen Unmasking Solution (Vector laboratories, Inc. Burlingame, CA). Sections were blocked using 10% goat serum and stained with anti- c-Kit (for ICC staining, Santa Cruz Biotechnology, Inc., Santa Cruz, CA, USA) antibody at 4°C overnight. Then, FITC-labeled anti-rabbit antibody was incubated with sections at room temperature for 1 hour. For neutrophil and macrophage staining, sections were incubated with FITC-anti-Ly-6C/G (a marker for neutrophil, Invitrogen Corp., Carlsbad, CA) or FITC-anti-F4/80 (a marker for macrophage, Invitrogen) antibody at 4°C overnight, respectively. Sections were mounted using Vectashield™ Mounting Medium containing DAPI (Vector laboratories, Inc. Burlingame, CA) and observed by fluorescence microscopy. FITC and DAPI images were taken from the same field.

The number of ICC per 20X power field and the numbers of neutrophil and macrophage per 10X power field were determined by counting the number of positive cells in at least 20 fields.

### Apoptotic assay

Apoptosis was detected in tissue sections using ApopTag™ *In Situ* Oligo Ligation (ISOL) kit (Intergen Company, Purchase, NY), following manufacturer’s guidelines, and observed by differential interference contrast (DIC) microscopy. The number of apoptotic cell per 40X power field was determined by counting the number of positive cells in at least 20 fields.

### Statistical analysis

Statistical significance of the difference between FJI and Roux-en-Y groups was determined using *student t test* analysis for nutritional status and *x*^*2*^*test* analysis *for* the incidence of postoperative complications. Statistical significance of cell numbers from beagle study was determined by one-way ANOVA followed by Newman-Keuls analysis using Prism 5.0 (GraphPad Software, Inc. San Diego, CA) for multiple comparisons. Data are presented as mean ± S.E.M. A *p* value < 0.05 was defined as statistically significant.

## Results

### Beneficial effects of FJI on the functional outcomes in gastric cancer patients after total gastrectomy

We evaluated post-operative nutritional status and complications up to 12 months post-operatively. In this study, all patients in FJI and Roux-en-Y groups survived for 12 months after surgery. First, we evaluated the PNI score, which correlates with the nutritional status in patients after surgery. PNI scores in patients receiving FJI (48.14 ± 5.22 at 3 months and 53.70 ± 5.83 at 12 months after operation) were significant higher than those in patients with Roux-en-Y (45.85 ± 5.59 at 3 months and 51.74 ± 5.49 at 12 months) (Table 2).

**Table 2:** **The PNI score in patients after total gastrectomy**

	**Roux-en-Y**	**FJI**	P value*
3 months	45.85 ± 5.59 (N = 79)	51.74 ± 5.49 (N = 71)	<0.05
12 months	48.14 ± 5.22 (N = 79)	53.70 ± 5.83 (N = 71)	<0.05

*Compared to PNI scores at the same post-operative time.

Then, we assessed the changes of bodyweight and blood hemoglobin and total protein levels in patients at 3 and 12 months after surgery. Compared to the bodyweight before surgery, the bodyweight loss at three and twelve months after surgery in the FJI groups with stage I-II and II-IV gastric cancer was significant less than that in patients with Roux-en-Y (Table 3). The increases of blood hemoglobin and total protein at three and twelve months after surgery, as compared to those at 1 month after surgery, were significantly higher in patients with stage III-IV gastric cancer receiving FJI than Roux-en-Y group (Table 3). Although, in patients with stage I-II disease, patients receiving FJI showed higher levels of increases of blood hemoglobin and total protein levels at three and twelve months after operation, as compared those in patients with Roux-en-Y, these changes were not significant (Table 3). These data suggested that retaining duodenal food transit in post-gastrectomy reconstruction supports good nutritional status, which may be more important for patients with late stage gastric cancers.

**Table 3:** **The nutritional status of patients after total gastrectomy**

**Time after surgery**	**3 months**		**12 months**	
**Disease stage**	**I-II**	**III-IV**	**I-II**	**III-IV**
Bodyweight loss (kg)^#^				
Roux-en-Y	6.03 ± 2.72	6.91 ± 1.84	5.36 ± 2.50	5.87 ± 2.17
FJI	5.36 ± 2.50	5.65 ± 1.72	3.11 ± 2.47	3.52 ± 2.18
*P* value*	0.01	0.036	0.01	0.041
Hemoglobin increase (g/dL)^**†**^				
Roux-en-Y	9.29 ± 5.76	5.92 ± 4.52	9.29 ± 5.76	7.02 ± 4.84
FJI	10.31 ± 5.91	8.97 ± 6.07	10.31 ± 5.91	10.00 ± 6.36
*P* value*	0.65	0.002	0.65	0.004
Total protein increase (g/dl)^**†**^				
Roux-en-Y	4.18 ± 2.59	4.08 ± 2.18	4.96 ± 3.06	5.59 ± 2.79
FJI	5.32 ± 2.75	5.52 ± 2.74	6.67 ± 3.35	7.32 ± 3.29
*P* value*	0.279	0.002	0.179	0.002

^#^Shown value = the value at the indicated post-operative time – the value before the surgery^#^ or the value at 1 month after the surgery^**†**^.

*Comparison was made between these two groups with the same disease stage and at the same post-operative time.

There are three common postoperative complications of reconstruction after total gastrectomy, reflux esophagitis, dumping syndrome, and Rous-en-Y syndrome. The incidence rates of reflux esophagitis, dumping syndrome, and Roux-en-Y syndrome in patients receiving Roux-en-Y were 16.5%, 16.5%, and 17.7%, respectively, which were significantly decreased to 4.2%, 5.6%, and 5.6% in patients receiving FJI, at 12 months after total gastrectomy (Table 4). Thus, the FJI procedure decreases post-operative complications in gastric cancer patients after total gastrectomy. Our data from patient study showed that FJI promotes nutritional recovery and decreases the risk of subsequent complications after gastrectomy, as compared to Roux-en-Y.

**Table 4:** **The incidence rates of postoperative complications at 12 months after total gastrectomy**

	**Roux-en-Y**	**FJI**	***Χ*** **2**	***P value***
Dumping Syndrome	16.5% (13/79)	5.6% (4/71)	6.437	0.040
Reflux Esophagitis	16.55 (13/79)	4.2% (3/71)	7.231	0.027
Roux-en-Y Syndrome	17.7% (14/79)	5.6% (4/71)	6.549	0.038

Furthermore, no class II and higher complications defined in Clavien-Dindo classification, such as anastomotic leakage, pancreatic fistula and bleeding, were found in patients receiving Roux-en-Y and FJI procedure in this study.

### FJI’s beneficial effects on ameliorating intestinal inflammation and damage and preserving intestinal motility in beagles

To investigate the physiologic mechanisms underlying FJI benefitting nutritional recovery and decreasing complications, we performed animal studies. We first studied the intestinal transit rate (the active carbon migration distance/the total small intestinal length) in beagles. At 48 hours postoperatively, the intestinal transit rate in sham group was 0.14 ± 0.03. Although the intestinal transit rate in FJI group (0.32 ± 0.11) was significantly higher than that in the sham group (p < 0.05), it was significant lower than that in Roux-en-Y group (0.52 ± 0.21, p < 0.05). These data suggested that FJI exerts a reservoir function to benefit food storage.

**Figure 2:**
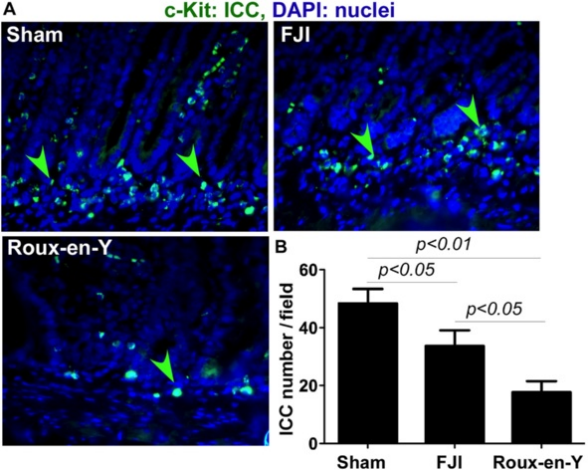
FJI preserves ICC in the small intestine. The intestinal tissues were isolated from beagles at 48 hours postoperatively. Paraffin-embedded tissue sections were stained using an anti-c-Kit (ICC marker) antibody and a FITC-conjugated secondary antibody. Nuclei were stained using DAPI (blue). Fluorescent and DAPI images were taken from the same field **(A)**. Green arrowheads: ICC. The number of ICC per 20X power field is shown **(B)**. Sham = 5 beagles, FJI = 10 beagles, Roux-en-Y = 7 beagles.

To study the effects of FJI and Roux-en-Y procedures on preserving intestinal motility, we detected interstitial cells of Cajal (ICC) in the intestine. Immunohistochemistry analysis of ICC showed that there were more ICC in submuocsa of the small intestine in FJI group than those in Roux-en-Y group (Figure 2A and B). Therefore, this result indicates that FJI preserves ICC, which may contribute to maintaining intestinal functional motility.

**Figure 3:**
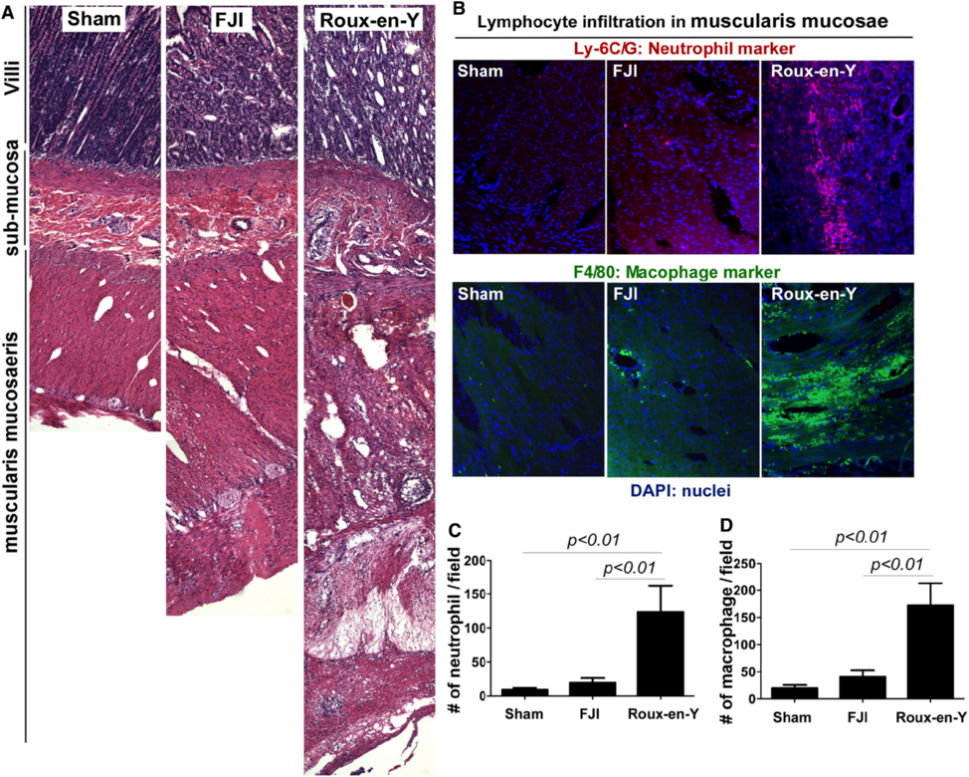
FJI decreases inflammation and neutrophil and macrophage infiltration into the small intestine in beagles. Beagle tissue sections were prepared as described in Figure 2. Tssue sections were stained using hematoxylin and eosin to assess injury and inflammation **(A)**. Neutrophil and macrophage were stained using Cy3-conjugated anti-Ly-6C/G (a marker for neutrophil) and FITC-conjugated anti-F4/80 (a marker for macrophage) antibodies, respectively. Nuclei were stained using DAPI (blue) **(B)**. The numbers of neutrophil **(C)** and macrophage **(D)** per 10X power field are shown. Sham = 5 beagles, FJI = 10 beagles, Roux-en-Y = 7 beagles.

Inflammation is a major factor to disrupt intestinal motility, including ICC network. Therefore, we evaluated the inflammatory responses after surgery. 60% (6/10) beagles receiving FJI and 100% (7/7) beagles receiving Roux-en-Y showed inflammation in serosal side of the small intestine around anastomosis, including hemorrhage, fibrin deposition, and ulceration (Figure 3A). The degree of serosal inflammation was lower in FJI, compared to that in Roux-en-Y group. Inflammatory responses after surgery include significant increase in lymphocyte infiltration and pro-inflammatory cytokine production. We found that the numbers of neutrophil and macrophage infiltration in muscularis mucosae of the small intestine were less in FJI group than that in Roux-en-Y group (Figure 3B-D). We did not find significant inflammation in mucosa of the small intestine in any of these three groups.

**Figure 4:**
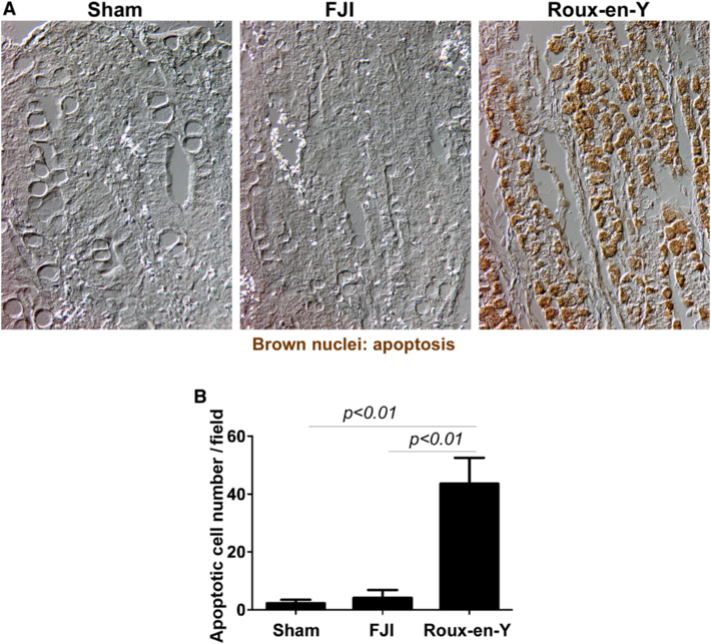
FJI decreases apoptosis in the small intestinal epithelial cells in beagles. Beagle tissue sections were prepared as described in Figure 2. Apoptosis was detected using ISOL kit (brown nuclei) **(A)**. The number of apoptotic cells per 40X power field is shown **(B)**. Sham = 5 beagles, FJI = 10 beagles, Roux-en-Y = 7 beagles.

Disruption of the integrity of this monolayer is a major defect in intestinal inflammation [11]. Thus, we performed an apoptotic assay to detect apoptosis in the intestine. We found that small intestinal epithelial cell apoptosis was significant reduced in beagles receiving FJI, compared to that in Roux-en-Y group (Figure 4A and B). These data indicate that FJI results in less intestinal tissue injury and inflammation when compared to Roux-en-Y.

## Discussion

Gastrointestinal surgery usually results in gastrointestinal tissue injury which leads to inflammatory responses within the tunica muscularis. The present study revealed that FJI benefits food storage and ameliorates intestinal inflammation and damage which may contribute to reducing ICC loss. ICC receives motor neural and mechanical inputs and generate and propagate electrical rhythmicity to control gastrointestinal motility [12-15]. ICC is responsible for generating and propagating electrical slow waves that coordinate the phasic contractions [15,16]. It has been reported that there were loss of acute disruption of ICC networks, slow waves, and phasic contractions five hours after surgery [17]. Thus, we detected ICC in the small intestinal 48 hours after operation. These beneficial effects of FJI may contribute to the improvement of outcomes after total gastrectomy in patients with gastric cancer observed in our clinical studies.

Many techniques for the restoration of intestinal continuity after gastrectomy have been proposed, both to compensate for the lost function of the stomach and in attempts to prevent postgastrectomy syndromes. The continuity of gastrointestinal tract plays a key role in the coordination of intestinal motility. Emerging evidence has shown that surgical manipulation of gastrointestinal tract, such as resection followed by reanastomosis, resulted in disruption of intestinal motility [18]. Roux-en-Y Syndrome represents a syndrome of abdominal pain, nausea, vomiting and fullness [19]. Roux-en-Y Syndrome has been frequently observed in patients who received Roux-en-Y reconstruction after partial gastrectomy with morbidity as higher as 30% [20]. It was suggested that the major cause of Roux-en-Y Syndrome was due to disruption of intestinal integrity and enteric neural continuity, leading to the subsequent disorder of intestinal motility. Furthermore, several studies found that disruption of the motility by gastrointestinal resection was through damage of ICC, the pacemakers for the gastrointestinal tract. Our study showed that FJI preserved ICC compared to Rou-en-Y procedure. Thus, FJI may exert the beneficial effects on reducing Roux-en-Y Syndrome.

The integrity of the intestinal epithelium is critical for maintaining normal intestinal functions while providing a defense against pathogenic microbes and detrimental substances in the intestinal lumen. The degree of inflammation in the muscularis is related to the degree of manipulation of the intestinal tissues. Inflammation may lead to several pathological responses, such as the loss of ICC proximal to intestinal obstructions after gastrointestinal surgery [21]. Therefore, reduced inflammation may mediate preservation of ICC in patients receiving FJI.

Chyme is mixed with bile and pancreatic secretions in duodenum. The passage of food triggers secreting of intestinal hormones such as secretin, cholecystokinin and insulin. Therefore, preserving duodenal food passage should enhance the digestive function. For example, duodenal food passage has been shown to exert beneficial effects on regulating blood sugar level after total gastrectom [3]. It has been reported that the type of reconstruction procedure after total gastrectomy plays a role in regulating postprandial gastrointestinal hormone production [22]. Therefore, facilitating food storage by FJI may contribute to its outcomes to improve nutritional status in patients.

In summary, FJI’s clinical beneficial outcomes may be associated with the reservoir function and its effects on preserving intestinal motility. Therefore, FJI has potential clinical application to improve the quality of life for patients after total gastrectomy.

## Conclusion

Our clinical studies indicate that the FJI procedure promotes nutritional recovery and decreases post-operative complications in patients with gastric cancer after total gastrectomy. Findings from animal studies further provide insights into understanding the physiologic mechanisms by which FJI exerts beneficial effects on outcomes after total gastrectomy, including preserving reservoir function and maintaining intestinal motility. Based on the surgical procedure used for FJI, FJI can maintain jejunal continuity and duodenal food passage. Thus, retaining the intestinal integrity by FJI may lead to beneficial clinical outcomes after surgery. However, more clinical studies are needed to further assess FJI to procedures using the conventional jejunal interposition.
